# Auditory and reward structures reflect the pleasure of musical expectancies during naturalistic listening

**DOI:** 10.3389/fnins.2023.1209398

**Published:** 2023-10-19

**Authors:** Benjamin P. Gold, Marcus T. Pearce, Anthony R. McIntosh, Catie Chang, Alain Dagher, Robert J. Zatorre

**Affiliations:** ^1^Department of Electrical and Computer Engineering, Vanderbilt University, Nashville, TN, United States; ^2^Vanderbilt University Institute of Imaging Science, Vanderbilt University Medical Center, Nashville, TN, United States; ^3^Montreal Neurological Institute, McGill University, Montreal, QC, Canada; ^4^International Laboratory for Brain, Music and Sound Research (BRAMS), Montreal, QC, Canada; ^5^Centre for Research on Brain, Language and Music (CRBLM), Montreal, QC, Canada; ^6^Centre for Interdisciplinary Research in Music, Media, and Technology (CIRMMT), Montreal, QC, Canada; ^7^Cognitive Science Research Group, School of Electronic Engineering & Computer Science, Queen Mary University of London, London, United Kingdom; ^8^Department of Clinical Medicine, Aarhus University, Aarhus, Denmark; ^9^Baycrest Centre, Rotman Research Institute, Toronto, ON, Canada; ^10^Department of Psychology, University of Toronto, Toronto, ON, Canada; ^11^Department of Biomedical Engineering, Vanderbilt University, Nashville, TN, United States; ^12^Department of Computer Science, Vanderbilt University, Nashville, TN, United States

**Keywords:** music, fMRI, pleasure, expectancies, ventral striatum (VS), superior temporal gyrus (STG)

## Abstract

Enjoying music consistently engages key structures of the neural auditory and reward systems such as the right superior temporal gyrus (R STG) and ventral striatum (VS). Expectations seem to play a central role in this effect, as preferences reliably vary according to listeners’ uncertainty about the musical future and surprise about the musical past. Accordingly, VS activity reflects the pleasure of musical surprise, and exhibits stronger correlations with R STG activity as pleasure grows. Yet the reward value of musical surprise – and thus the reason for these surprises engaging the reward system – remains an open question. Recent models of predictive neural processing and learning suggest that forming, testing, and updating hypotheses about one’s environment may be intrinsically rewarding, and that the constantly evolving structure of musical patterns could provide ample opportunity for this procedure. Consistent with these accounts, our group previously found that listeners tend to prefer melodic excerpts taken from real music when it either validates their uncertain melodic predictions (i.e., is high in uncertainty and low in surprise) or when it challenges their highly confident ones (i.e., is low in uncertainty and high in surprise). An independent research group (Cheung et al., 2019) replicated these results with musical chord sequences, and identified their fMRI correlates in the STG, amygdala, and hippocampus but not the VS, raising new questions about the neural mechanisms of musical pleasure that the present study seeks to address. Here, we assessed concurrent liking ratings and hemodynamic fMRI signals as 24 participants listened to 50 naturalistic, real-world musical excerpts that varied across wide spectra of computationally modeled uncertainty and surprise. As in previous studies, liking ratings exhibited an interaction between uncertainty and surprise, with the strongest preferences for high uncertainty/low surprise and low uncertainty/high surprise. FMRI results also replicated previous findings, with music liking effects in the R STG and VS. Furthermore, we identify interactions between uncertainty and surprise on the one hand, and liking and surprise on the other, in VS activity. Altogether, these results provide important support for the hypothesized role of the VS in deriving pleasure from learning about musical structure.

## 1. Introduction

Enjoying music is one of the most universally and consistently rewarding human pleasures (Dubé and Le Bel, 2003). Expectation seems to play an important role in this experience (Sloboda, 1991; Grewe et al., 2007; Salimpoor et al., 2011; Cheung et al., 2019; Shany et al., 2019), as music’s ever-changing structure is particularly well suited to elicit predictions, attention, and surprise (Meyer, 1956; Huron, 2006; Zald and Zatorre, 2011; Gebauer et al., 2012; Koelsch et al., 2019; Vuust et al., 2022). Even after repeated exposures, when listeners may have more (but not full) understanding of a stimulus, musical complexity significantly modulates liking (Smith and Cuddy, 1986; North et al., 2000; Madison and Schiölde, 2017; Gold et al., 2019b).

Neuroimaging studies provide valuable insights about the biological mechanisms underlying the relationship between musical expectations and pleasure. A recent meta-analysis of 38 studies identified a robust association between music liking and neural activity in the ventral striatum (VS), anterior prefrontal cortex (aPFC), and right superior temporal gyrus (R STG) (Mas-Herrero et al., 2021b), which comprise key structures of the reward and auditory processing networks (Zatorre et al., 2002; Haber and Knutson, 2010). The most prominent relationship was in the ventral striatum, where music liking ratings correlate with linear increases in hemodynamic response (Blood and Zatorre, 2001; Salimpoor et al., 2013; Martínez-Molina et al., 2016) and dopaminergic activity (Salimpoor et al., 2011). The striatum, and especially the nucleus accumbens (NAc) that is found in its ventral aspect, is a central hub of the neural reward system, and receives inputs from the prefrontal cortex, subcortical limbic regions, and dopaminergic midbrain nuclei (Haber and Knutson, 2010). With these connections, the NAc integrates affective and goal-oriented information for action selection (Floresco, 2015). Perhaps most notably, the NAc is the primary site of reward prediction errors (RPEs) as measured in rodents, monkeys, and humans: changes in endogenous activity that signal the difference between the expected and experienced value of goal-oriented events (Schultz et al., 1997; Rutledge et al., 2010; Chase et al., 2015; Floresco, 2015). RPEs in the ventral striatum could therefore account for much of the pleasure associated with musical expectation and surprise, as we and others have hypothesized (Zald and Zatorre, 2011; Gebauer et al., 2012; Zatorre and Salimpoor, 2013; Salimpoor et al., 2015).

Our team previously tested this RPE-based hypothesis for music (Gold et al., 2019a) with a paradigm and computational model common in the global RPE literature (Watkins, 1989; Sutton and Barto, 1998; Gläscher et al., 2010; Daw et al., 2011). Specifically, and since research on RPEs has largely focused on action selection, in our prior study participants chose between arbitrary alternatives during music listening (e.g., blue vs. yellow) that were associated with different probabilities of the music ending pleasantly vs. unpleasantly, based on the ongoing context (Gold et al., 2019a). While the participants were never told about these probabilities explicitly, their choices indicated learning of the contingencies throughout the task. At the same time, functional magnetic resonance imaging (fMRI) data indicated that blood oxygen level-dependent (BOLD) responses in the NAc reflected computationally modeled RPEs and were linked to successful task completion, providing compelling evidence of RPEs. This finding therefore demonstrated that an aesthetic stimulus like music can elicit RPEs, just like – and in the same context as – the more concrete rewards previously used in these experiments, such as money, food, or juice. Yet while those other rewards offer clear adaptive benefits such as nutrition or the means to procure it, the biological value of music remains unclear.

One possible explanation of music’s value is the reward of learning itself, and especially learning to improve one’s predictions (Vuust and Kringelbach, 2010; Zald and Zatorre, 2011; Gebauer et al., 2012; Koelsch et al., 2019; Vander Elst et al., 2021). Humans and non-human animals are willing to sacrifice food and money to learn non-instrumental information – i.e., that which they cannot necessarily use to gain future rewards – such as answers to trivia questions or the probability that a predetermined reward is *en route* (Bromberg-Martin and Hikosaka, 2009; Kang et al., 2009; Bennett et al., 2016; Brydevall et al., 2018). Receiving this information activates the ventral striatum and dopamine release therein (Kang et al., 2009; Bromberg-Martin and Hikosaka, 2011; Jepma et al., 2012; Ripollés et al., 2014; Brydevall et al., 2018). Meanwhile, as patterns of rhythm, melody, harmony, and other features unfold and evolve over time, they facilitate the generation, evaluation, and updating of predictions – often with opportunities to test those updated predictions in the next musical phrase, chorus, movement, etc. (Meyer, 1956; Huron, 2006; Zald and Zatorre, 2011; Gebauer et al., 2012). Several studies show that humans automatically and implicitly learn about musical structure and make predictions based on it (Loui and Wessel, 2007, 2008; Loui et al., 2010; Hansen and Pearce, 2014; Barascud et al., 2016), even from birth (Partanen et al., 2013; Virtala et al., 2013). The pleasure of music listening may therefore derive at least in part from rewards associated with learning about musical structure, commensurate with ventral striatal activity and dopamine transmission.

The aesthetic pleasure of learning is not a new concept, although it has received renewed attention in the last few years (Franklin and Adams, 2011; Salimpoor et al., 2011, 2013; Van de Cruys and Wagemans, 2011; Chmiel and Schubert, 2017; Wassiliwizky et al., 2017). In the 19th century, Wilhelm Wundt hypothesized that stimuli of intermediate complexity, uncertainty, or novelty were more arousing, rewarding, and pleasurable (Wundt, 1874). In the 1960s and ‘70s, Daniel Berlyne identified this hypothesized “Wundt effect” in terms of an inverted U-shaped relationship between measures of complexity (e.g., predictability, surprise, uncertainty, or familiarity) and liking (Berlyne, 1971, 1974). Since then, a wealth of cross-disciplinary research has found similar inverted U-shaped relationships between complexity on the one hand, and attention, curiosity, motivation, and learning on the other (Kang et al., 2009; Abuhamdeh and Csikszentmihalyi, 2012a,b; Gottlieb et al., 2013; Baranes et al., 2015; Oudeyer et al., 2016; Chmiel and Schubert, 2017; Brydevall et al., 2018). Music may therefore modulate learning, and thus the reward thereof, according to its complexity (Smith and Cuddy, 1986; North et al., 2000; Madison and Schiölde, 2017).

To examine this possibility, in a previous study our team used a well-validated model of musical informatics (Pearce, 2005, 2018) to quantify the complexity of a variety of real-world musical melodies, both in terms of information content (i.e., surprise) and entropy (i.e., predictive uncertainty), and compared these measures to liking ratings (Gold et al., 2019b). As hypothesized, we found inverted U-shaped relationships between complexity and liking, which persisted across several repetitions. We also identified an interaction between information content and entropy, such that listeners reliably preferred music that was relatively low in one of these features and high in the other. In other words, and in keeping with models of prediction-based learning (Friston, 2005, 2010; Pearce, 2005; O’Reilly, 2013; Koelsch et al., 2019; Vuust et al., 2022), listeners preferred surprising events when their uncertainty was low (informing them that they did, in fact, have more to learn), and unsurprising ones when their uncertainty was high (signaling that their uncertain predictions were, in fact, correct). These findings support the idea that learning about musical structure is intrinsically rewarding, and may account for the neural reward system’s involvement in musical pleasure.

Using a similar approach, Cheung et al. (2019) modeled the information content and entropy of chord sequences from real-world pop music, and evaluated both the liking ratings and BOLD activity associated with each chord during fMRI. Like our team, these authors found that information content and entropy had interacting effects on liking ratings, with the highest ratings reserved for uncertain and surprising or highly certain and unsurprising chords. In addition, their neuroimaging analyses implicated the superior temporal gyrus (STG) and medial temporal lobe (i.e., amygdala and hippocampus) in this interaction. Yet while ventral striatal activity covaried with entropy, it did not reflect significant effects of information content or the interaction between them, reigniting questions about why this structure and its dopamine transmission so robustly correlate with musical pleasure (*cf.* Blood and Zatorre, 2001; Salimpoor et al., 2011; Martínez-Molina et al., 2016; Gold et al., 2019a; Mas-Herrero et al., 2021a,b).

In the context of these questions, the present study has three primary aims. First, we assess the replicability of previously reported relationships between liking ratings, musical information content, and entropy, and hypothesize that we will find an interaction similar to those in Cheung et al. (2019) and Gold et al. (2019b). Second, we use fMRI to investigate whether the brain regions implicated in musical pleasure show different responses for liked vs. disliked surprises during naturalistic music listening, consistent with our group’s prior work on action-oriented RPEs. And third, we evaluate how neural activity (indexed with fMRI BOLD signals) reflects behavioral information content × entropy interactions. While Cheung et al. (2019) also examined information content × entropy interactions with fMRI, our study differs from theirs in that we draw on real-world musical excerpts of different genres and time periods that vary widely in surprise and uncertainty, operationalize information content and entropy for entire melodic excerpts rather than individual chords, and relate the BOLD responses for musical complexity to music liking. With this approach, we expect to find evidence of information content × entropy effects in brain regions associated with musical pleasure.

## 2. Materials and methods

The participants, stimuli, information-theoretic modeling, experimental procedure, and data acquisition were previously described in Gold (2019). Analysis scripts are available at https://github.com/benjaminpgold/auditory-reward-pleasure-of-musical-expectancies.git.

### 2.1. Participants

This study was approved by the Research Ethics Board of the Montreal Neurological Institute. Screening questionnaires ensured that participants were neurologically healthy, had normal hearing, had not participated in previous experiments with the stimuli (Gold et al., 2019b), and were eligible to undergo safe fMRI scanning. We also confirmed that all participants listened to Western tonal music and that their musical expectations were not largely based on atonal or jazz music, which frequently deviate from the structures of Western folk and classical music we used to train our computational model, by asking the volunteers’ five favorite genres; none of them listed any atonal or jazz genres. The sample size was 24 (17 females, 7 males, mean age ± standard deviation = 22.08 ± 2.70 years).

Nine participants had formal musical experience, but only two were still actively playing music (whole-group mean musical experience ± standard deviation = 2.89 ± 4.52 years). These participants placed at approximately the 33rd percentile of normative Goldsmiths Musical Sophistication Index scores (Müllensiefen et al., 2014) (mean total score ± standard deviation = 72.78 ± 20.96), and within typical ranges on the Barcelona Music Reward Questionnaire (Mas-Herrero et al., 2013) (mean total score ± standard deviation = 81.39 ± 10.80).

### 2.2. Stimuli

We used 50 experimental stimuli, plus two for practice trials. All of these were excerpts of real, pre-composed music collected from public Musical Instrument Digital Interface (MIDI) databases, most of them from the following websites: www4.osk.3web.ne.jp/~kasumitu/eng.htm, www.classicalarchives.com/midi.html, and www.baldwinsmusic.com. Each of these stimuli were also used in our previous research (Gold et al., 2019b), and contained examples of several musical genres from a wide range of time periods, composers, tonalities, and meters (Supplementary Table S1). We used only monophonic stimuli (i.e., containing only one tone at a time) to avoid the confounding effects of harmony (i.e., chordal relationships) and polyphony (i.e., multiple voices), and we reduced other confounds by normalizing the peak amplitudes to the same level with Audacity® (© 1999–2018 Audacity Team), limiting the stimuli to 30 ± 2 s, and synthesizing the MIDI stimuli into Waveform Audio File (WAV) format. We also standardized the tempo of each stimulus to either 96, 120, or 144 bpm – whichever sounded most musically appropriate – with MuseScore (© 2018 MuseScore BVPA). These considerations constrained our stimuli to excerpts that were either solo pieces or melodic lines from polyphonic pieces.

We converted these stimuli into naturalistic-sounding WAV files with the Kontakt 5 synthesizer (© 2018 Native Instruments GmbH) within the Ableton Live 9 digital audio workstation (© 2018 Ableton). We generated each excerpt with a flute digital synthesizer, digitally filtered them to resemble the acoustics of a music studio, and randomly shifted the note onsets on the order of milliseconds using Ableton’s Groove Pool with 25% randomization for “humanization” – i.e., to prevent the stimuli from sounding mechanical and unnatural.

### 2.3. Information-theoretic modeling

Across many different experimental paradigms and musical samples, the Information Dynamics of Music (IDyOM) model has been shown to provide reliable measures of musical unpredictability/surprise (as represented by information content) and uncertainty (as represented by entropy) in Western listeners (Pearce, 2005, 2018; Pearce and Wiggins, 2006; Pearce et al., 2010a; Omigie et al., 2012; Egermann et al., 2013; Hansen and Pearce, 2014; Sauvé et al., 2018). IDyOM has significantly outperformed similar models and explained up to 83% of the variance in listeners’ pitch expectations (Pearce, 2005, 2018; Pearce et al., 2010b; Hansen and Pearce, 2014), while also successfully predicting several electrophysiological, psychophysiological, and subjective emotional responses (Carrus et al., 2013; Egermann et al., 2013; Omigie et al., 2013; Sauvé et al., 2018).

We used IDyOM to operationalize the amount of surprise and uncertainty in our stimuli, following the same procedure as in our group’s previous work (see Gold et al., 2019b for details). This approach involved training an IDyOM model on a large corpus of Western tonal music to simulate how humans learn musical statistics through long-term exposure and also on each stimulus to simulate how humans learn about the statistical properties of each individual piece. Given this training, our IDyOM model calculated the information content and entropy of each note, derived from probability distributions of its pitch and inter-onset interval in context. We then computed the weighted mean information content and entropy for each stimulus by multiplying its note-by-note measures by their relative duration and averaging these duration-weighted values, again following the procedure outlined in Gold et al. (2019b). Throughout the manuscript, we refer to these measures as mDW-IC (mean duration-weighted information content) and mDW-Entropy (mean duration-weighted entropy).

### 2.4. Experimental procedure

During scanning (see below), participants listened to each 30-s MIDI stimulus using MRI-compatible S14 Insert Earphones (Sensimetrics Corporation, Malden, MA), pre-set to a comfortable volume, while a fixation cross appeared on the screen *via* an angled mirror on top of the MRI head coil. The task was conducted with Presentation® software (Neurobehavioral Systems, Inc., Berkeley, CA). As they listened, the participants rated how much they liked the music, in real time, with an MRI-compatible Legacy Joystick in their right hand (Current Designs, Inc., Philadelphia, PA). The joystick position ranged from −150 to 150, sampled at 1000 Hz. They were instructed that they should be rating their moment-by-moment liking whenever music was playing, but that they would only see the fixation cross throughout the experiment to avoid vision-related confounds in the functional MRI signal. The time between stimuli was randomly jittered between 5.5 and 6.5 s to allow for separable hemodynamic responses and to prevent participants from successfully predicting when stimuli would begin.

Before the experimental task, participants experienced three practice trials using stimuli that did not occur during the experiment for familiarization and to ensure that they understood the instructions. During the first two practice trials, the participants could see a vertical bar for their ratings, labeled “not at all” on the bottom and “very much” at the top, with a cursor that moved as the joystick did. In the third practice trial, which repeated the same stimulus as the first one, the bar and cursor were no longer visible and participants only saw a fixation cross on the screen.

To avoid anchoring effects, we sorted the stimuli into five clusters of mDW-IC using Matlab’s k-means clustering algorithm, and randomly selected one stimulus from each cluster to constitute the first five stimuli of the experiment so that the participants could acclimate to the range of stimuli present in the rest of the experiment. After these five, the remaining 45 stimuli occurred in a random and participant-specific order.

Shortly after they had left the scanner, participants rated their prior familiarity with the experimental stimuli, also *via* Presentation software, with one of the following options: “I did not know any of them; I knew about 1–10 of them; I knew about 11–20 of them; I knew about half of them; I knew about 30–40 of them; I know almost all of them.” Thirteen participants said “I did not know any of them” and eleven said “I knew about 1–10 of them,” showing that the stimuli were for the most part unfamiliar.

### 2.5. FMRI data acquisition and preprocessing

We acquired MRI data with a Siemens MAGNETOM Prisma fit 3 T scanner and a 32-channel head coil at the McConnell Brain Imaging Centre of the Montreal Neurological Institute. We used a T2*-weighted multi-band echo planar imaging sequence to collect whole-brain functional blood oxygen level-dependent (BOLD) images at high temporal resolution (54 slices, TE = 30 ms, TR = 654 ms, multi-band acceleration factor = 6, flip angle = 60°, matrix size = 210 × 210 × 135, voxel size = 2.5 mm isotropic). We also collected reversed phase-encode scans to acquire pairs of images with susceptibility-induced distortions in opposite directions and estimate the off-resonance field of our T2*-weighted images (Andersson et al., 2003), and then unwarped our volumes using FSL’s topup command (Smith et al., 2004). When whole-brain coverage was not possible with these parameters, we prioritized ventral temporal and frontal regions at the cost of dorsal parietal ones. The experimental task occurred over two functional runs of 25 trials and about 17 min each, with a short break in between for rest, except for one participant who opted to end the experiment during the 49th trial. We therefore discarded this participant’s data for the 49th and 50th trials.

We also collected a high-resolution T1-weighted image for each participant (MPRAGE: TE = 2.98 ms, TR = 2,300 ms, matrix size = 256 × 256 × 192, voxel size = 1 mm isotropic) for anatomical registration. We preprocessed the functional images with FSL FEAT Version 6 (FMRIB, UK), starting with discarding the first 6 volumes (3.924 s) of each run to accommodate scanner drift. Then, following the procedure outlined in Pruim et al. (2015), we performed motion correction and calculated movement regressors (with FSL MCFLIRT), 4D mean intensity normalization, and spatial smoothing (with a Gaussian 5-mm FWHM kernel) before using ICA-based Automatic Removal of Motion Artifacts (ICA-AROMA) to clean the data of motion-related artifacts. We then removed linear trends and effects of white-matter and cerebrospinal-fluid signals by regressing these out of the 4D data, and high-pass filtered the resulting time series with Gaussian-weighted least-squares straight-line fitting sigma = 50 s. Finally, we used FSL FLIRT with 12 DOF to linearly register each functional run to the participant’s T1-weighted space, and FSL FNIRT with 10-mm warp resolution to non-linearly normalize the volumes to the MNI152 2-mm standard brain. Reported coordinates are in Montreal Neurological Institute space.

### 2.6. Behavioral analyses

As in our team’s previous work (Gold et al., 2019b), and in Cheung et al. (2019), we used linear mixed-effects models to detect group-level effects that arise from the trial-by-trial and individual-by-individual variability. Using Matlab’s fitlme function and the procedure recommended in Diggle et al. (2002) and Zuur et al. (2009), we first optimized the random-effects structure of a “beyond-optimal” model (including all relevant fixed effects and interactions) according to the Akaike information criterion *via* restricted maximum likelihood estimation, identifying and incorporating any differences between participants (e.g., their baseline liking) that could help explain their ratings. We then optimized the fixed-effects structure *via* likelihood ratio tests of nested models and Akaike information criterion of other models using maximum likelihood estimation, and finally evaluated the model with restricted maximum likelihood estimation. Separate mixed-effects models evaluated the main effects of mDW-IC (i.e., surprise), mDW-Entropy (i.e., uncertainty), and the interaction between them, using *z*-scored values of these variables to allow for comparisons between their linear and quadratic effects.

While linear mixed-effects modeling is a powerful tool for probing interactions such as that between mDW-IC and mDW-Entropy, visualizing the results of this approach is often difficult due to the intercepts and/or slopes that vary across individual participants. We thus present the estimated responses of the best-fitting models, as in prior studies (e.g., Cheung et al., 2019; Gold et al., 2019b). Yet to aid the interpretability of the complex interactions between mDW-IC and mDW-Entropy on liking ratings, we also supplemented our linear mixed-effects model by categorizing stimuli for simpler visualization. Specifically, and following our previous procedure (Gold et al., 2019b), we classified each stimulus according to its mDW-Entropy and mDW-IC using Matlab’s k-means clustering algorithm to obtain data-driven stimulus categories. Starting with six points roughly corresponding to stimuli of low or high mDW-Entropy and low, medium, or high mDW-IC (see Supplementary Figure S1), this algorithm identified six stimulus clusters through Euclidean distance minimization without any information about the participants’ liking ratings. We then assessed the mean and standard error of the average liking ratings for each of these categories and plotted the results.

### 2.7. FMRI analyses

We tested the effects of mDW-IC, mDW-Entropy, and liking by generating subject-specific parametric regressors of the mDW-IC, mDW-Entropy, and average liking rating for each stimulus, each normalized from −1 to 1 to accommodate individual differences in the ranges of the ratings used and enable comparisons across regressors and participants. We then created parametric interaction regressors through element-wise multiplication: i.e., by multiplying the normalized mDW-IC value for each stimulus by its normalized mDW-Entropy value (for the interaction between surprise and uncertainty) or by its subject-specific, normalized liking value (for the interaction between surprise and liking). To control for the effects of musical onsets and music vs. silence, we also created binary spike regressors for the beginning and binary music regressors for the duration of each stimulus. Since the mDW-IC, mDW-Entropy, average liking, and interaction regressors were all parametric modulations of the music vs. silence regressor, which in turn covaried with the musical onsets regressor, we orthogonalized each of these with respect to the binary music vs. silence and musical onsets terms.

We convolved all of these regressors with a canonical hemodynamic response function and its temporal derivatives to account for temporal variations. We then conducted first-level general linear models (GLMs) for each run of each participant. In addition to the aforementioned regressors of musical onsets and music vs. silence, the models we evaluated consisted of:

1. BOLD as a function of Liking.
2. BOLD as a function of mDW-IC.
3. BOLD as a function of mDW-Entropy.
4. BOLD as a function of (mDW-IC × Liking).
5. BOLD as a function of (mDW-IC × mDW-Entropy).

Since interaction effects may sometimes arise from only one of the interacting variables, we also conducted parallel GLMs for these relationships. In the first one, we assessed BOLD as a function of (mDW-IC × Liking) + mDW-IC + Liking, with the latter two regressors orthogonalized with respect to the first. In the second one, we examined BOLD as a function of (mDW-IC × mDW-Entropy) + mDW-IC + mDW-Entropy, with the latter two regressors orthogonalized with respect to the first.

These GLMs also included 24 motion regressors to account for movement-related variance: one for each directional axis, one for each derivative of each axis, and the squares of these 12 values. We also scrubbed volumes with framewise displacement above 0.9 with additional nuisance regressors (*cf.* Siegel et al., 2014). These motion-related regressors were not convolved.

We then conducted second-level fixed-effects analyses for each participant to average the contrast estimates of each participant’s two runs, and group-level mixed-effects analyses to evaluate whole-group results. For visualization and transparency, we present these group effects for the whole brain at an uncorrected threshold of *z* ≥ 2. For statistical analyses, we evaluated effects in three *a priori* regions of interest (ROIs) defined by a meta-analysis of music-specific reward correlates (Mas-Herrero et al., 2021b). For each of these ROIs – which centered on the ventral striatum (VS), right superior temporal gyrus (R STG), and anterior prefrontal cortex (aPFC) – we extracted the average beta values for each regressor of interest and conducted two-tailed *t-*tests to assess their difference from a null effect of 0. For the main effect of liking, we also tested whether participants’ average, non-normalized liking ratings throughout the task (i.e., their overall or ‘baseline’ music liking) covaried with the BOLD activity in any of the *a priori* ROIs. We report uncorrected *p-*values and those corrected with the Benjamini–Hochberg false discovery rate (FDR) procedure.

Since the liking ratings reflected movements of a joystick, and the striatum plays a well-documented role in motor control, we assessed whether any liking effects in the *a priori* VS ROI could be attributed to movement rather than liking *per se*. To do so, we extracted the mean preprocessed BOLD signal of the ROI for each trial, as well as the variance of the joystick rating time series (after *z*-scoring the ratings for each participant) and the mean and variance of the root mean square absolute head movement (derived from FSL MCFLIRT). We also computed the Pearson’s correlation between the VS BOLD data and head motion time series for each trial. We then fit linear mixed-effects models to investigate the relationships between these features across trials, scanning runs, and participants, determining the random effects structures as in Diggle et al. (2002) and Zuur et al. (2009) and corrected the *p-*values of these tests for multiple comparisons. These models consisted of:

1. Mean VS BOLD activity as a function of joystick movement (i.e., Liking rating variance).
2. Mean head movement as a function of joystick movement.
3. Mean VS BOLD activity as a function of mean head movement.
4. Mean VS BOLD activity as a function of head movement variance.
5. VS BOLD-head motion correlation as a function of joystick movement.

To facilitate the visualization and interpretation of interaction effects (mDW-IC × Liking and mDW-IC × mDW-Entropy), we separated the stimuli into distinct categories and plotted the BOLD responses associated with each, as in our categorical analysis of the behavioral mDW-IC × mDW-Entropy interaction described above. However, instead of the six categories we used to analyze the behavioral interaction, we generated just four categories for the fMRI interactions to prevent the noisiness of BOLD data from leading to undersampled clusters.

Since liking ratings are inherently subjective, we generated subject-specific categories to interrogate the mDW-IC × Liking interaction. Specifically, we normalized each participant’s liking ratings to range from −1 to 1 (matching the range of the normalized mDW-IC values), and then categorized stimuli with normalized mDW-IC ≤ 0 and subject-specific liking ≤0 as low mDW-IC and low liking, those with normalized mDW-IC ≤ 0 and subject-specific liking >0 as low mDW-IC and high liking, etc. We then conducted a GLM with binary regressors of these categories and the onset spike regressor, evaluated the participants’ average BOLD responses for these four stimulus categories, and assessed the significance of these categorical interactions with repeated-measures ANOVAs in each *a priori* ROI (including terms for the main effects of mDW-IC and Liking). We report uncorrected and FDR-corrected *p* values. To better understand the interaction effect and accommodate for the noisiness of BOLD data along with minor registration and/or activation peak anomalies, we also examined categorical responses in activation clusters from the original (non-categorical) interaction GLM (thresholded at uncorrected *p* ≤ 0.01) that overlapped with the *a priori* ROIs and visualized the significant result(s).

Since the mDW-IC and mDW-Entropy values for each stimulus are not subject-dependent, we used k-means clustering to categorize the stimuli for follow-up analyses of the mDW-IC × mDW-Entropy interaction (Supplementary Figure S1). We then conducted GLMs with a binary regressor for each of the four categories and the onset spike regressor, skipping the binary regressor for the duration of each stimulus since it would simply be the sum of the four category regressors. Next, we evaluated the BOLD responses for these categories in each *a priori* ROI and activation cluster from the original GLM of this interaction (thresholded at uncorrected *p* ≤ 0.01) that overlapped with them (including terms for the main effects of mDW-IC and mDW-Entropy) to visualize the interaction effects. We report uncorrected and FDR-corrected *p-*values.

## 3. Results

### 3.1. Liking ratings replicate the interaction between musical surprise and uncertainty

**Figure 1:**
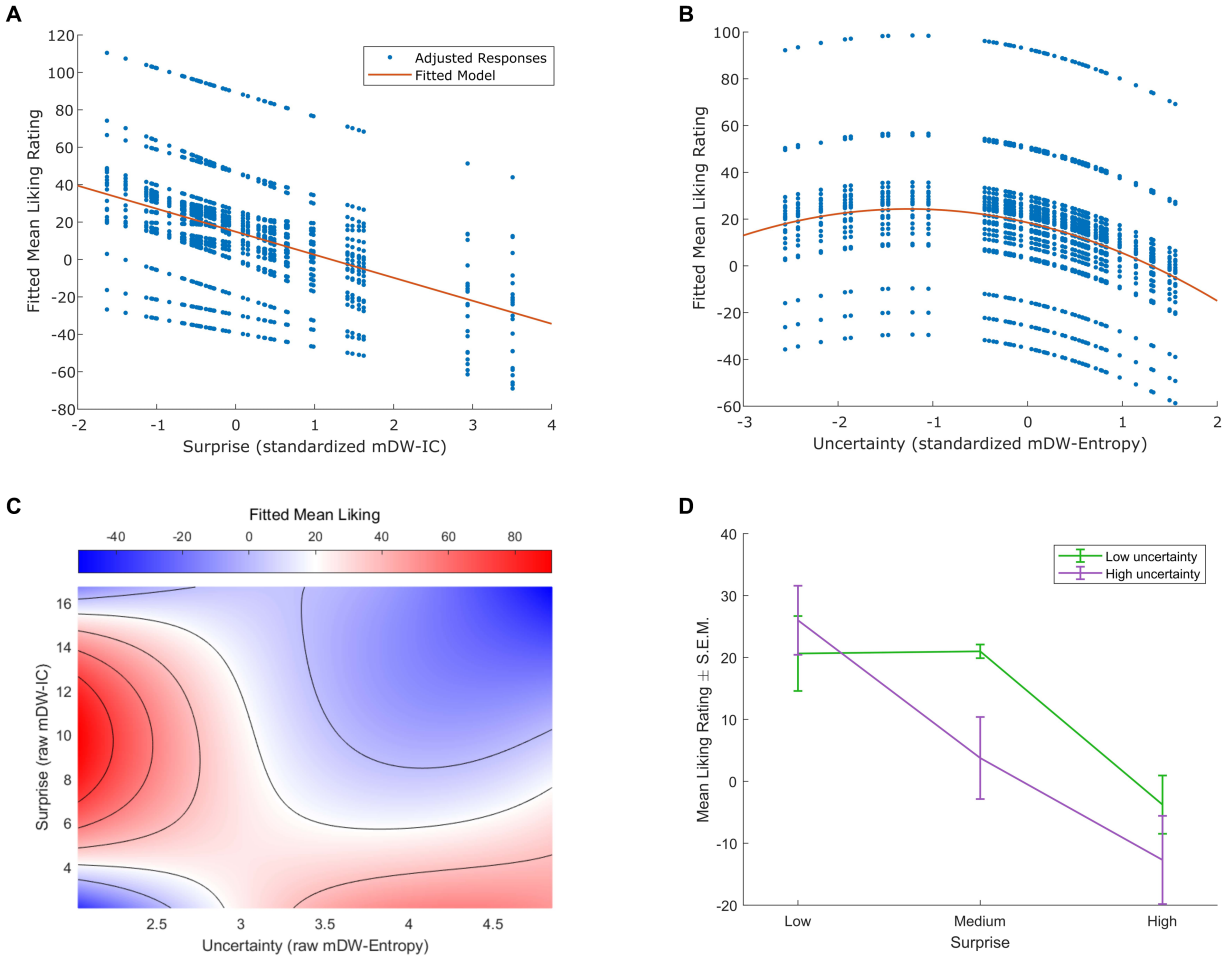
Effects of musical surprise and uncertainty on liking. **(A)** Linear mixed-effects modeling indicated a significant negative linear relationship between surprise (measured as standardized mean duration-weighted information content, or mDW-IC) and liking ratings (*p* < 0.001). The best-fitting model had subject-varying intercepts and slopes, but only the model-adjusted data points (in blue) and trend line (in red) are shown here for clarity. **(B)** The best-fitting linear mixed-effects model of uncertainty (measured as standardized mean duration-weighted entropy, or mDW-Entropy) and liking ratings indicated negative linear and quadratic terms consistent with a Wundt effect (*p* < 0.001). This model contained subject-varying intercepts and slopes, but only the model-adjusted data points (in blue) and trend line (in red) are shown here for clarity. **(C)** Linear mixed-effects modeling of interactions between surprise (raw, i.e., unstandardized mDW-IC) and uncertainty (raw mDW-Entropy) on liking ratings revealed a saddle-shaped effect with higher ratings for stimuli of low uncertainty and intermediate surprise or those of high uncertainty and low surprise (*p* < 0.001). The best-fitting model contained subject-varying intercepts and slopes for the effect of surprise, but only the model-predicted ratings are shown here for clarity, using a blue-to-red color scale. **(D)** Categorizing the stimuli with three levels of mDW-IC and two levels of mDW-Entropy helped to illustrate the interaction between surprise and uncertainty on liking ratings. The mean liking ratings ± the standard error of the mean (S.E.M.) are shown here for each cluster of stimuli.

The best-fitting model of the relationship between surprise (mDW-IC) and liking ratings expressed a negative linear relationship (Figure 1A) with subject-varying intercepts (95% CI = 19.06, 34.54) and slopes (95% CI = 4.05, 9.64). This model explained 46.2% of the variance in liking ratings (*p* < 0.001) with its negative linear coefficient (*β* = −12.30, *p* < 0.001). An alternative model that added a quadratic effect of surprise on liking ratings was also significant (*p* < 0.001), but its quadratic term was not (linear *β* = −11.32, *p* < 0.001; quadratic *β* = −0.70, *p* = 0.223). Accordingly, this alternative model did not fit liking ratings better than the one with only a linear term [likelihood ratio test *χ^2^*(1, *N* = 24) = 1.48, *p* = 0.223].

For the relationship between uncertainty (mDW-Entropy) and liking, the best-fitting model indicated an inverted U-shaped Wundt effect (Figure 1B). This model explained 38.4% of the variance in liking ratings (*p* < 0.001) and contained a negative linear term (*β* = −9.30, *p* < 0.001), a negative quadratic term (*β* = −3.71, *p* < 0.001), and random effects of participant intercepts (95% CI = 7.96, 29.06), uncertainty slopes (95% CI = −11.71, −6.89), and uncertainty^2^ slopes (95% CI = −5.29, −2.13). Alternative models with only linear or quadratic fixed-effects terms yielded significantly worse fits of liking ratings, as indicated by likelihood ratio tests [linear + quadratic model vs. linear-only model *χ^2^*(1, *N* = 24) = 21.05, *p* < 0.001; linear + quadratic model vs. quadratic-only model *χ^2^*(1, *N* = 24) = 56.10, *p* < 0.001].

We also observed a significant interaction between surprise (mDW-IC) and uncertainty (mDW-Entropy) on liking ratings. The best-fitting linear mixed-effects model of this interaction revealed a saddle-shaped effect wherein participants preferred stimuli of low uncertainty and intermediate surprise or those of high uncertainty and low surprise (Figure 1C). This model explained 49.6% of the variance in liking ratings (*p* < 0.001) and contained linear and quadratic terms for both surprise and uncertainty, as well as their interactions, as well as random intercepts (95% CI = 22.73, 43.72) and surprise slopes (95% CI = 1.50, 3.48) across participants. Categorizing each stimulus into bins of low or high uncertainty and low, medium, or high surprise also showed the greatest ratings for stimulus clusters of high uncertainty and low surprise or low uncertainty and low-to-intermediate surprise (Figure 1D) in a pattern that closely resembles prior findings (Gold et al., 2019b).

### 3.2. Normalized liking ratings correlate with R STG responses, while average liking correlates with VS activity

**Figure 2:**
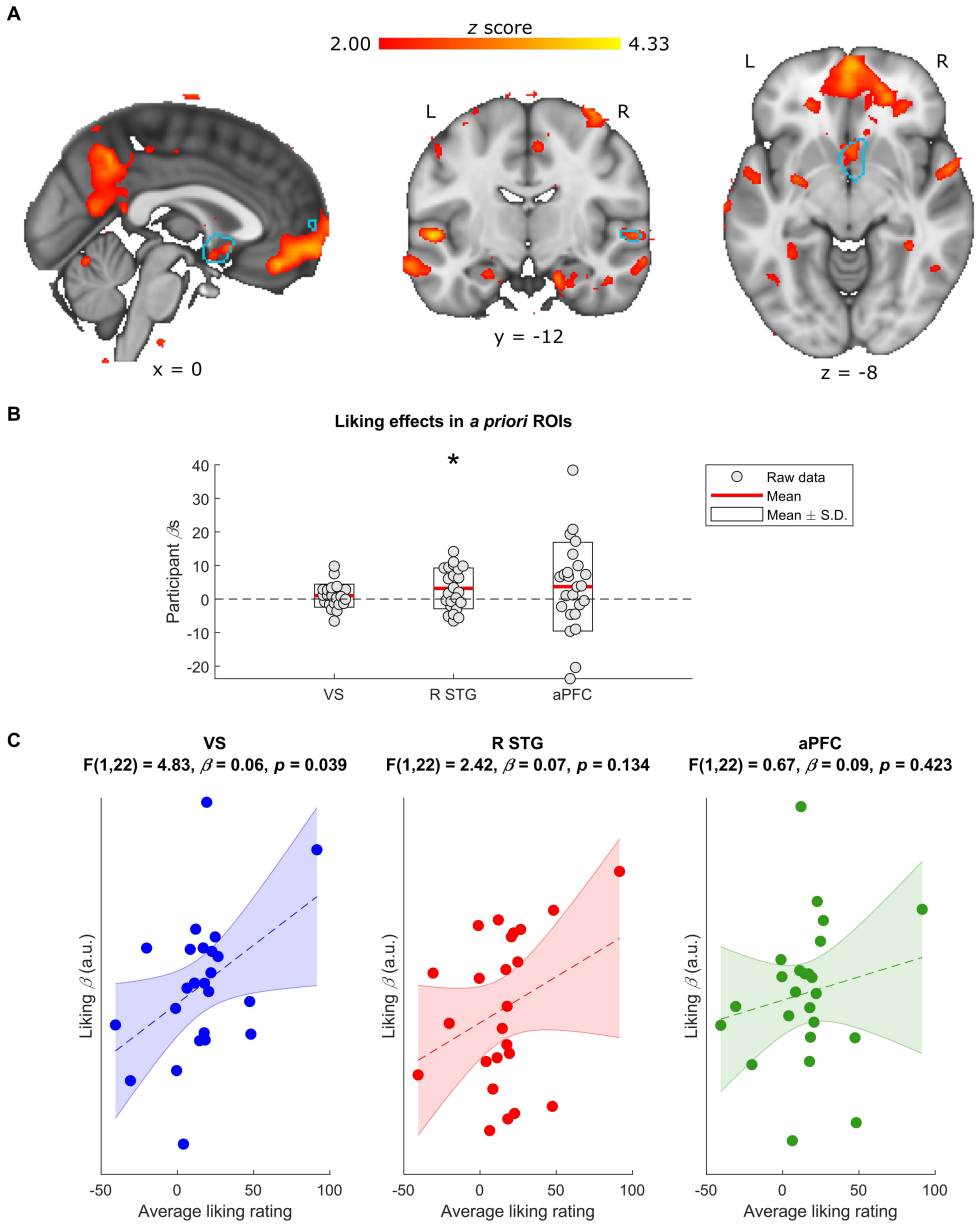
Neural effects of liking ratings. **(A)** Whole-group normalized liking ratings covaried with BOLD activity in a number of regions, including the R STG and the VS. Data are visualized in MNI space at an uncorrected threshold of *z* ≥ 2 in a red-to-yellow color scale, while the *a priori* regions of interest (ROIs) from Mas-Herrero et al. (2021b) are outlined in light blue. **(B)** Extracting the BOLD effects for each *a priori* ROI revealed a positive effect of normalized liking ratings on R STG activity (uncorrected *p* = 0.018) but not VS or aPFC activity (uncorrected *p*s ≥ 0.166). Individual-participant *β*s are shown in grey dots, while the mean for each ROI is shown with red lines and the mean ± standard deviation (S.D.) of each distribution is shown in white boxes. **(C)** Participants’ average liking ratings for all stimuli significantly covarsied with their BOLD responses to liking (i.e., liking *β*) in the VS (uncorrected *p* = 0.039) but not in the R STG or aPFC (uncorrected *p*s ≥ 0.135). Data are visualized as dots with the liking *β*s in arbitrary units (a.u.), along with the line of best fit (in dashes) and its standard error (shaded) for the linear regression between average liking ratings and liking *β*s in each ROI. L, left; R, right; VS, ventral striatum; R STG, right superior temporal gyrus; aPFC, anterior prefrontal cortex. * Uncorrected *p* < 0.05.

Before investigating how neural responses to musical complexity related to music liking, we first assessed the main effect of liking ratings on our sample’s BOLD activity. This analysis suggested that changes in normalized liking ratings significantly covaried with changes in the BOLD signals of the R STG *a priori* ROI [*t*(23) = 2.56, uncorrected *p* = 0.018, FDR-corrected *p* = 0.053], while effects in the VS (uncorrected *p* = 0.166) and aPFC (uncorrected *p* = 0.185) failed to reach significance (Figures 2A,B).

Though normalizing liking ratings reduces inter-individual variance, some of that variance may be informative: i.e., reflecting differences in the participants’ overall or baseline liking of the stimuli. We therefore compared each individual’s BOLD responses to music liking (i.e., liking *β*) with their average liking rating throughout the task. These linear regressions revealed an effect in the *a priori* VS ROI [*F*(1,22) = 4.83, *β* = 0.06, uncorrected *p* = 0.039, FDR-corrected *p* = 0.116] such that participants who tended to rate the stimuli higher had stronger liking-related responses in this region (Figure 2C). Though excluding the participant with the highest overall liking weakened this result (uncorrected *p* = 0.271), reducing outlier effects with robust regression validated the original finding [*F*(1,22) = 4.92, *β* = 0.05, uncorrected *p* = 0.037, FDR-corrected *p* = 0.112]. Effects in the *a priori* R STG and aPFC ROIs were not significant (uncorrected *p*s ≥ 0.134).

Since the participants in this study registered their liking ratings by moving a joystick, and the striatum in particular plays a major role in movement, we probed the above results to determine whether they could be explained by motor activity rather than liking. Specifically, we extracted the BOLD signals from the *a priori* VS ROI in each trial and evaluated whether movements of the head or joystick correlated with these signals. Linear mixed-effects models indicated that the variance of liking ratings (i.e., the extent of joystick manipulation) was not correlated with the VS BOLD response (uncorrected *p* = 0.139) but did exhibit a slight relationship with the amount of head motion in a trial (*β* = 0.03, uncorrected *p* = 0.021, FDR-corrected *p* = 0.105). Yet VS BOLD activity was not significantly affected by the mean (uncorrected *p* = 0.831) or variance (uncorrected *p* = 0.094) of head movement, and the relationship between VS BOLD signals and head motion was not related to the variance of joystick movement (uncorrected *p* = 0.880). The above effects of ventral striatal BOLD signals are not therefore attributable to motion.

### 3.3. The VS reflects an interaction between surprise and liking

**Figure 3:**
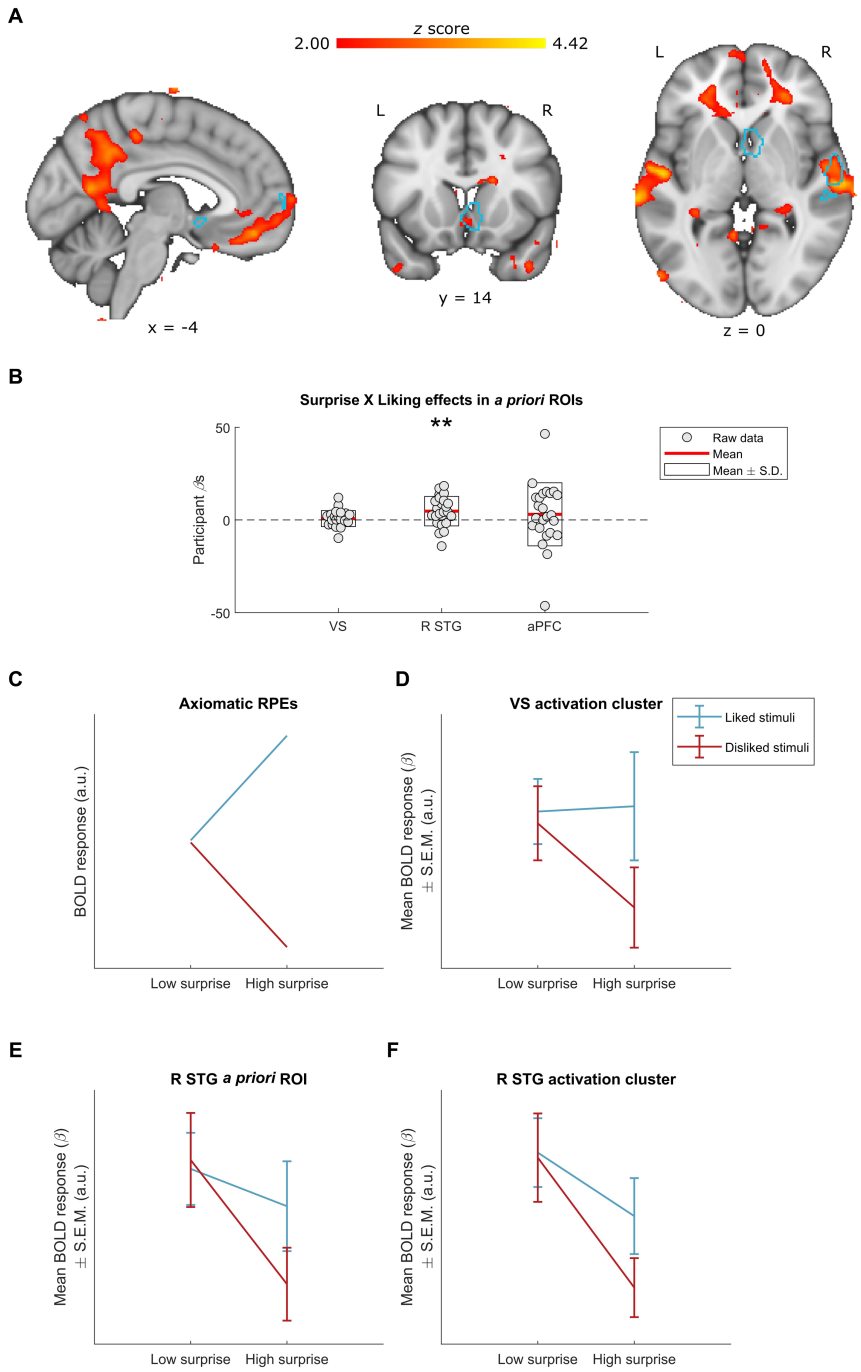
Neural effects of surprise × liking interactions. **(A)** Whole-group interactions between surprise (operationalized as mean duration-weighted information content, or mDW-IC) and liking ratings covaried with BOLD activity in a number of regions, including the R STG and the VS. Data are visualized in MNI space at an uncorrected threshold of *z* ≥ 2 in a red-to-yellow color scale, while the *a priori* regions of interest (ROIs) from Mas-Herrero et al. (2021b) are outlined in light blue. **(B)** Extracting the BOLD effects for each *a priori* ROI revealed a significant effect in the R STG activity (uncorrected *p* = 0.008) but not VS or aPFC activity (uncorrected *p*s ≥ 0.392). Individual-participant *β*s are shown in grey dots, while the mean for each ROI is shown with red lines and the mean ± standard deviation (S.D.) of each distribution is shown in white boxes. **(C–F)** Dividing the stimuli into liked vs. disliked excerpts at two levels of surprise allowed for further inspection of this interaction. **(C)** We illustrate how BOLD responses for each stimulus category would look if following the axiomatic properties of reward prediction errors (RPEs), in arbitrary units (a.u.) (see Rutledge et al., 2010; Fouragnan et al., 2017). **(D)** The activation cluster in the VS (i.e., the red-yellow cluster overlapping with the light-blue outline in the coronal slice from **A**) exhibited a pattern of responses resembling the axiomatic properties of RPEs: i.e., similar activity for liked vs. disliked stimuli of low surprise, greater activity for liked stimuli of high surprise, and lesser activity for disliked stimuli of high surprise. Data are visualized as mean BOLD responses ± the standard error of the mean (S.E.M.) in arbitrary units (a.u.). **(E)** The interaction in the *a priori* R STG also exhibited similar activity for liked vs. disliked stimuli of low surprise, but with decreasing responses for high surprise regardless of liking. Data are visualized as mean BOLD responses ± the standard error of the mean (S.E.M.) in arbitrary units (a.u.). **(F)** The activation cluster in the R STG (i.e., the red-yellow cluster overlapping with the light-blue outline in the axial slice from panel **A**) exhibited a pattern of responses highly similar to that of the *a priori* R STG ROI. Data are visualized as mean BOLD responses ± the standard error of the mean (S.E.M.) in arbitrary units (a.u.). L, left; R, right; VS, ventral striatum; R STG, right superior temporal gyrus; aPFC, anterior prefrontal cortex. ** Uncorrected *p* < 0.01.

The interaction between musical surprise (mDW-IC) and liking significantly engaged the R STG ROI [*t*(23) = 2.92, uncorrected *p* = 0.008, FDR-corrected *p* = 0.023], with non-significant effects in the VS (uncorrected *p* = 0.409) and aPFC (uncorrected *p* = 0.392) (Figures 3A,B). Each *a priori* ROI exhibited overlap with an activation cluster, though, and so we examined these clusters as well as the *a priori* ROIs in follow-up analyses. A parallel GLM that included covariates for the main effects of surprise and liking yielded highly similar results (Supplementary Figure S2).

To better understand these interactions, we divided the stimuli into four categories according to their mDW-IC value and subject-specific liking rating (see Materials and Methods). Where the GLM indicated a significant effect, we then visualized the average BOLD responses for each category to discern the nature of their interaction. Finally, we qualitatively compared the patterns of these categorical BOLD responses to an axiomatic model of reward prediction errors (RPEs), which indicates increasing responses for more surprising and liked stimuli and decreasing responses for more surprising and disliked ones (see Rutledge et al., 2010; Fouragnan et al., 2017; Figure 3C).

This approach revealed that the BOLD activity in the *a priori* R STG ROI (Figure 3E) and the activation cluster that overlapped with it (Figure 3F) followed the same pattern of responses across the four stimulus categories, with more surprising stimuli eliciting less activity especially when they were also disliked. Conversely, the ventral striatal cluster overlapping with the *a priori* ROI was the only one that resembled the axiomatic pattern (Figure 3D), suggesting that this region might exhibit RPEs during music listening (*cf.* Shany et al., 2019; Gold et al., 2019a).

### 3.4. Surprise and uncertainty interact in VS BOLD responses, following a different pattern than behavioral surprise × uncertainty effects

**Figure 4:**
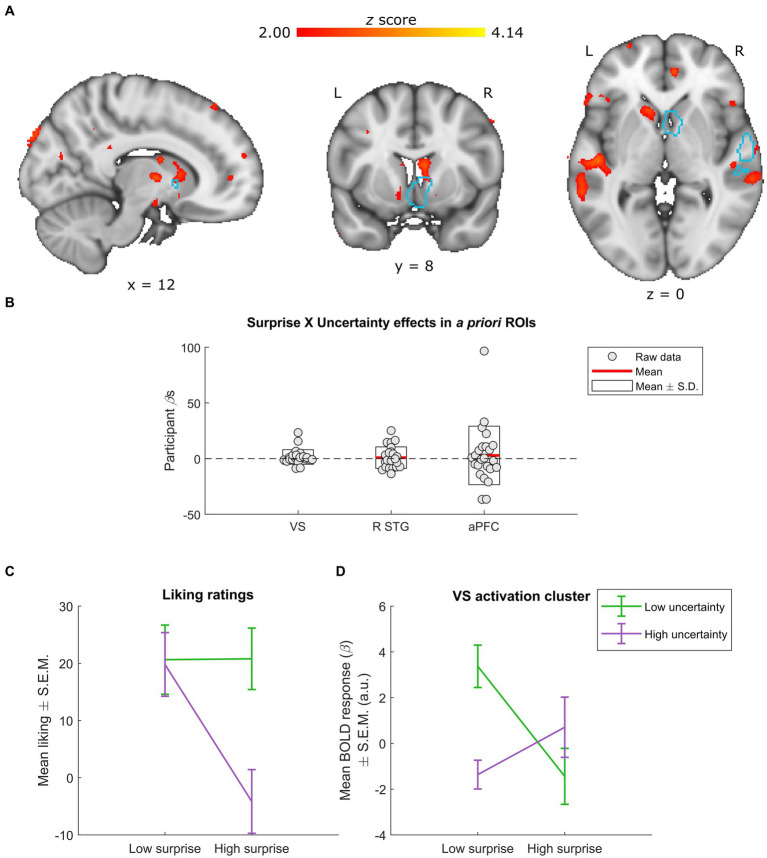
Neural effects of surprise × uncertainty interactions. **(A)** Whole-group interactions between surprise (operationalized as mean duration-weighted information content, or mDW-IC) and uncertainty (operationalized as mean duration-weighted entropy, or mDW-Entropy) covaried with BOLD activity in a number of regions, including the VS. Data are visualized in MNI space at an uncorrected threshold of *z* ≥ 2 in a red-to-yellow color scale, while the *a priori* regions of interest (ROIs) from Mas-Herrero et al. (2021b) are outlined in light blue. **(B)** Extracting the BOLD effects for each *a priori* ROI revealed no significant effects (uncorrected *p*s ≥ 0.297). Individual-participant *β*s are shown in grey dots, while the mean for each ROI is shown with red lines and the mean ± standard deviation (S.D.) of each distribution is shown in white boxes. **(C,D)** Dividing the stimuli into two levels of surprise and two levels of uncertainty allowed for further inspection of this interaction. **(C)** Across four categories (as opposed to the six used in Figure 1D), liking ratings exhibit relatively similar responses for stimuli with low uncertainty (regardless of surprise) and those with high uncertainty and low surprise. In contrast, stimuli with high uncertainty and high surprise receive much lower average liking ratings. Data are visualized as mean liking ratings ± the standard error of the mean (S.E.M.). **(D)** The activation cluster in the VS (i.e., the red-yellow cluster overlapping with the light-blue outline in the coronal slice from panel **A**) exhibited a crossover effect, with low uncertainty yielding more activity for low vs. high surprise and high uncertainty yielding more activity for high vs. low surprise. Data are visualized as mean BOLD responses ± the standard error of the mean (S.E.M.) in arbitrary units (a.u.).

The interaction between surprise (mDW-IC) and uncertainty (mDW-Entropy) did not reach significance in the *a priori* ROIs (uncorrected *p*s ≥ 0.297), and only the VS ROI overlapped with a subthreshold activation cluster (Figures 4A,B). A parallel analysis that included covariates for the main effects of surprise and uncertainty yielded a highly similar pattern of results (Supplementary Figure S3).

As in the above surprise × liking analysis, we divided the stimuli into four categories to characterize the shape of any interactions. For comparisons to the behavioral results, we also recalculated the average liking ratings for the surprise × uncertainty interaction across these four stimulus categories (Figure 4C). This procedure indicated that none of the *a priori* ROIs or overlapping activation clusters had patterns of responses that resembled the behavioral results. Instead, the interaction in the cluster overlapping the *a priori* VS ROI illustrated a crossover effect, with lesser activity for stimuli with low uncertainty and high surprise or high uncertainty and low surprise (Figure 4D).

We also evaluated main effects of surprise (mDW-IC) and uncertainty (mDW-Entropy) for completeness. These results are shown in Supplementary Figure S4.

## 4. Discussion

Prominent models of music enjoyment point to a central role of expectancy in the psychological, neural, and psychophysiological processes underlying pleasure (Meyer, 1956; Huron, 2006; Zald and Zatorre, 2011; Gebauer et al., 2012; Zatorre and Salimpoor, 2013; Salimpoor et al., 2015; Koelsch et al., 2019; Vuust et al., 2022). These models are based on music’s ability to establish and manipulate patterns; the occurrence of peak emotional, autonomic, and neural responses surrounding dramatic changes in musical structure; and the involvement of the brain’s reward system – which signals the value of anticipated and surprising events – in musical pleasure (Sloboda, 1991; Blood et al., 1999; Blood and Zatorre, 2001; Steinbeis et al., 2006; Grewe et al., 2007; Salimpoor et al., 2011, 2013; Egermann et al., 2013; Mas-Herrero et al., 2021b). However, evidence that directly and specifically associates the neural processing of musical expectancies to reward structures or pleasure is quite limited.

The present study therefore seeks to link musical expectancies to both pleasure and its neural substrates. Using a naturalistic music listening paradigm and information-theoretic modeling during fMRI scanning, we replicate previous reports of music liking varying with musical uncertainty and surprise, and then extend this research to implicate the reward system in these effects. We also test the widely held hypothesis that the ventral striatum integrates information on musical reward and surprise, as in reward prediction errors (RPEs), providing crucial evidence reflecting this phenomenon during naturalistic listening.

### 4.1. Musical uncertainty and surprise jointly affect liking ratings

Our behavioral results align with previous reports of interactions between musical uncertainty and surprise on liking. We find this interaction using a mixed-effects linear model (Figure 1C), like Cheung et al. (2019), and further visualize it with stimulus clusters (Figure 1D), like Gold et al. (2019b). Also like in these previous studies, this interaction stems from preferences for music with high uncertainty and low surprise or low uncertainty and medium surprise, supporting models that attribute pleasure to either validating an uncertain model of one’s environment (in this case, music) or adding new information to a model strong enough to accommodate it (*cf.* Koelsch et al., 2019; Vuust et al., 2022).

Our interaction effect differs from that of Cheung et al. (2019), however, in that ours is based on ~30-s melodies from multiple eras and genres (Supplementary Table S1) while theirs was based on 2.4-s chords arranged in the progressions of late 20th-century pop songs. Our stimuli thus contain higher levels of uncertainty and surprise: see Table 1. In fact, we find that liking ratings decline for our most surprising stimuli even when uncertainty is low, while Cheung et al. only report greater liking for more surprising music during low uncertainty. This subtle difference could reflect a contrast between melodic vs. harmonic music processing, or the existence of a threshold for the pleasure of surprise. Future studies should investigate harmonic complexity and liking with a wider range of stimuli.

**Table 1:** Comparison of musical complexity features in the present stimuli and those of Cheung et al. (2019).

	**Information content**		**Entropy**	
	**Event level**	**Stimulus average**	**Event level**	**Stimulus average**
Gold et al. (present study)	0.21 – 38.29	2.66 – 17.19	1.19 – 10.50	3.97 – 5.36
Cheung et al. (2019)	0.05 – 22.53	3.57 – 6.96	0.23 – 7.79	2.34 – 3.86

Calculations for the information content and entropy values in the present are described in the Materials and Methods, though note that the values listed here are not duration-weighted to facilitate comparison. Information content and entropy values for the Cheung et al. (2019) stimuli come directly from that manuscript, which also describes the procedure for deriving them. Cells indicate the range of information content or entropy values for each event (note or chord) in the experiment or for the average values obtained from each stimulus.

Analyzing the main effect of uncertainty reveals an inverted U-shaped Wundt effect on liking ratings (Figure 1B), consistent with many models and reports of liking intermediate complexity in musical and other domains (Kang et al., 2009; Abuhamdeh and Csikszentmihalyi, 2012a,b; Witek et al., 2014; Baranes et al., 2015; Chmiel and Schubert, 2017; Brydevall et al., 2018; Matthews et al., 2019; Gold et al., 2019b). Again, these models posit that the draw of intermediate complexity is its ability to optimize arousal, attention, curiosity, and learning (Berlyne, 1971, 1974; Gottlieb et al., 2013; Kidd and Hayden, 2015; Oudeyer et al., 2016; Koelsch et al., 2019).

Yet surprise exhibits a linear effect on liking ratings, with liking decreasing as surprise increases (Figure 1A). At first glance, this result contrasts with our group’s prior finding of a quadratic, Wundt effect between these variables (Gold et al., 2019b). However, that effect was characterized by both linear and quadratic terms, such that more surprising stimuli evoked decreasing liking ratings in a curvilinear fashion – consistent with the linear relationship that we observe here. In both the present and our group’s previous studies, our choice to use real-world musical excerpts for ecological validity may have prevented us from observing stronger effects of the initial, rising portion of an inverted U-shaped effect, since sampling a sufficient range of complexity is a common issue in assessing Wundt effects that often leads to quadratic shapes appearing linear (Chmiel and Schubert, 2017). In the present study as opposed to our group’s previous one, the background noise of the MR scanner may have exacerbated this issue by contributing to a more unpleasant and entropic environment. Given the interactions between uncertainty and surprise, this noise might have thus driven preferences towards less surprising stimuli, resulting in a linear rather than quadratic effect. Future research on music complexity processing and liking in different environments can help elucidate this finding.

### 4.2. Interactions between musical surprise and liking are observable in neural activity during naturalistic listening

Analyzing the neural correlates of interactions between surprise and liking reveals effects in both the VS and R STG (Figure 3). These results indicate that neural auditory and reward regions are engaged in not just music liking but also how liking interacts with musical surprise in naturalistic listening. Further inspection suggests that these interactions arise from greater differences between low and high surprise responses among disliked stimuli.

These interactions conform with models that implicate reward prediction errors (RPEs) in musical pleasure, since RPEs similarly involve the joint processing of value and surprise (Schultz et al., 1997; Rutledge et al., 2010; Chase et al., 2015; Floresco, 2015). RPEs are closely linked with learning to maximize rewards and minimize punishments, and are most robustly found in dopamine and BOLD signals in the VS (Schultz et al., 1997; Rutledge et al., 2010; Chase et al., 2015; Floresco, 2015). Accordingly, we and others have hypothesized that music’s ability to manipulate expectations, induce strong feelings of reward and pleasure, and evoke VS activity – especially at moments of dramatic changes in musical structure – may be due to RPE computation (Zald and Zatorre, 2011; Gebauer et al., 2012; Zatorre and Salimpoor, 2013; Salimpoor et al., 2015). Our group previously investigated this possibility by adapting a commonly used decision-making task to musical contingencies and modeling the musical rewards, predictions, and errors that arose (Gold et al., 2019a). This research demonstrated that music could elicit RPEs in the VS, but did so in a context more suited to understanding action selection than musical pleasure.

In contrast, the present results suggest that musical surprises may tap into neural mechanisms of reward even during ecologically valid, naturalistic music listening. These results also complement those of the only other study, to our knowledge, that has directly examined the neural correlates of the interaction between musical surprise and liking. Using expert musicians to annotate moments of surprise, Shany et al. (2019) found that BOLD activity in the VS reflected a surprise × liking interaction. However, this effect was driven by large responses for surprises during highly liked music, with no indication that BOLD responses decreased for surprising and disliked music. Our findings thus provide the first evidence of a formally modeled surprise × liking interaction, and specifically one in the VS that follows the pattern of responses in RPEs. Accordingly, and as Shany et al. also posited, our results are consistent with the hypothesis that implicit expectations about musical events and structure may ultimately give rise to RPEs.

Nonetheless, it remains unclear how *sensory* expectations and surprises regarding, e.g., what note is heard at what time, relate to *reward* values and prediction errors in music. Emerging evidence suggests that midbrain dopamine neurons encode both sensory and reward prediction errors, contributing to the idea that learning about one’s environment may be intrinsically rewarding (see, e.g., Bromberg-Martin et al., 2010; Bromberg-Martin and Hikosaka, 2011; Keiflin and Janak, 2017; Sharpe et al., 2017). Meanwhile, listening to music often seems to lead to automatic and implicit learning of its structure (Loui and Wessel, 2007, 2008; Loui et al., 2010; Hansen and Pearce, 2014; Barascud et al., 2016). Better recollection of musical pieces, indicative of learning, is also associated with greater pleasure during listening – unless dopaminergic signaling is dampened (reviewed in Ferreri and Rodriguez-Fornells, 2022). These findings suggest that musical reward and pleasure may at least in part arise from gaining information about musical structure, i.e., sensory events (see, e.g., Vuust and Kringelbach, 2010; Zald and Zatorre, 2011; Gebauer et al., 2012; Koelsch et al., 2019; Vander Elst et al., 2021). While more research is needed to uncover the precise processes through which sensory predictability and surprise confer reward value, the interactions that we observe between musical surprise and liking in auditory and reward structures provide support for these regions’ hypothesized roles in linking musical expectancies to pleasure (Zald and Zatorre, 2011; Gebauer et al., 2012; Zatorre and Salimpoor, 2013; Salimpoor et al., 2015; Koelsch et al., 2019; Vander Elst et al., 2021).

### 4.3. The ventral striatum also reflects interacting uncertainty and surprise, in an unexpected pattern

FMRI data also indicate an interaction between uncertainty and surprise on BOLD activity in the striatum (Figures 4A,D). Although Cheung et al. (2019) did not find evidence of interacting harmonic uncertainty and surprise in this region, its strong association with both musical pleasure (Blood and Zatorre, 2001; Salimpoor et al., 2011, 2013; Martínez-Molina et al., 2016; Mas-Herrero et al., 2021b) and predictive processing (Schultz et al., 1997; Rutledge et al., 2010; Chase et al., 2015; Floresco, 2015; Fouragnan et al., 2017; Shany et al., 2019; Gold et al., 2019a) led us to hypothesize that its activity profile would reflect the processing underlying the behavioral interaction. However, the shape of this effect did not closely resemble the behavioral one (Figures 4C,D). Specifically, stimuli low in uncertainty and high in surprise elicited relatively high liking ratings but low BOLD responses, while those with high degrees of both uncertainty and surprise exhibited low liking ratings but the second-highest BOLD activity. Moreover, this effect arose in a relatively dorsal portion of the striatum that extends into the caudate, which has been associated with the anticipation of rewards – including peak musical pleasure – as opposed to the experience of pleasure *per se* (Haber and Knutson, 2010; Salimpoor et al., 2011). The interaction that we observe here might thus reflect a different aspect of musical complexity processing than the experience of pleasure.

### 4.4. Limitations

The present study has some important limitations. Most notably, our fMRI results have relatively small effect sizes that could betray type I errors rather than true effects. Adjusting significance levels these results to reduce potential type I errors thus decreased the number of effects deemed significant. There are several possible explanations for these limited effect sizes. One is that our short, monophonic, and mostly classical stimuli in the flute timbre did not elicit strong, naturalistic hedonic feelings, dampening the experience of pleasure and its relationship to complexity that we investigated. Another possibility is that our information-theoretic model and/or its parameters may not fully capture the hemodynamics of deriving pleasure from musical complexity. As this model evaluates musical features note-by-note, it may not be the best tool for studying relatively slow BOLD signals over the course of an entire melody or piece.

Collinearity between model terms is also likely to reduce effect sizes in interaction analyses. For example, we find a strong, linear relationship between musical surprise and liking (Figure 1A). This relationship differs from previous reports of inverted U-shaped effects of musical surprise on liking out of scanning contexts, including one that used the same stimuli as the present study (Chmiel and Schubert, 2017; Cheung et al., 2019; Gold et al., 2019b). As suggested above, the noise and relative discomfort of being in the MR scanner may have shifted participants’ preferences towards stimuli with fewer and smaller surprises. Whatever the reason, though, this correlation between surprise and liking limits our power to determine how surprise and liking interact in terms of BOLD activity.

Uncertainty and surprise are also closely related, both conceptually and in the variables we use to operationalize them here (Pearson’s *r* = 0.48, *p* < 0.001). Though we identify some interactions between them, consistent with other studies (Cheung et al., 2019; Gold et al., 2019b), their correlation again limits our power to distinguish these effects from type I errors. The noisiness of BOLD data also hampers statistical power, which could make interactions between uncertainty and surprise especially difficult to pinpoint with fMRI. Moreover, while these stimulus features are orthogonal in several contexts, music may not be one of them: i.e., auditory sequences with uncorrelated surprise and uncertainty might not be perceived or processed as “music.” Determining the extent to which these features are separable in music, and identifying/developing stimuli that separate them, will be an important step in further dissecting their independent and interacting effects. Doing so will be especially challenging when averaging across several musical events, as stimuli with more surprise on average are consequently more uncertain. More time-resolved analyses that capture the temporal dynamics between musical surprise and uncertainty might thus be better suited to differentiating their effects.

In fact, the temporal dynamics of music play a large role in its capacity for emotion and pleasure (Salimpoor et al., 2011; Gebauer et al., 2012; Pearce and Wiggins, 2012; Koelsch et al., 2019; Vander Elst et al., 2021). Averaging measures like surprise, uncertainty, and liking across a musical stimulus could therefore obfuscate some degree of their effects and interactions. For example, Mueller et al. (2015) report that ventral striatal responses to music decline after roughly 10 s. Fortunately, models like IDyOM can provide reliable estimates of note-by-note surprise and uncertainty (Pearce, 2005), while on-line ratings can capture participants’ responses to musical events as they occur. On the other hand, the high degree of variability in both the speed and the extent of raters’ reactions makes it difficult to associate on-line ratings with the event(s) that led to them or to compare such ratings across individuals. While some studies have handled these concerns with limited and well-defined scales (e.g., a rating range of 1–4 where 1 means “no pleasure,” 2 means “low pleasure,” etc., see Salimpoor et al., 2009, 2011; Mas-Herrero et al., 2021a), the much broader range of available ratings in the present study limited the interpretability and comparability of time-resolved joystick movements. Moreover, a supplementary analysis of the effect of liking on neural responses indicates that modeling the entirety (i.e., ~30 s) or just the onset (i.e., the first ms) of a stimulus yields highly similar results (Supplementary Figure S5). Modeling the first 10 s of a stimulus yields a slightly different pattern of activity, but doing so also overlooks the changes in liking, uncertainty, and surprise that occur past this window.

## 5. Conclusion

Despite these limitations, our findings lend new support to current models of musical processing and pleasure. We replicate behavioral evidence of an important role for predictive complexity in music liking, and present new evidence that links the pleasure of processing musical complexity directly to auditory and reward structures during naturalistic listening. In one analysis, we find an interaction between musical uncertainty and surprise in the striatum that supports this region’s hypothesized role in deriving pleasure from learning about musical structure. In another, we find an interaction between surprise and pleasure that supports the involvement of reward prediction errors in musical pleasure by suggesting that the striatum conveys reward signals based on musical expectations. These findings illustrate that melodic as well as harmonic complexity (*cf.* Cheung et al., 2019) is relevant for musical enjoyment and auditory-reward processing, and brings us closer to understanding what exactly makes music so pleasurable.

## Data availability statement

The raw data supporting the conclusions of this article will be made available by the authors, without undue reservation.

## Ethics statement

The studies involving humans were approved by The Research Ethics Board of the Montreal Neurological Institute. The studies were conducted in accordance with the local legislation and institutional requirements. The participants provided their written informed consent to participate in this study.

## Author contributions

BG, MP, AD, and RZ contributed to the conception and design of the study. BG analyzed the data with input from MP, AM, CC, AD, and RZ. BG wrote the first draft of the manuscript. All authors contributed to the article and approved the submitted version.

## Funding

This research was funded via a grant from the Canadian Institutes of Health Research to RJZ, and by funding to the McConnell Brain Imaging Centre from the Canada Brain Research Fund. RJZ is funded through the Canada Research Chair program and the Canadian Institute for Advanced Research. BPG was funded by a Collaborative Research and Training Experience in Complex Dynamics from the a Natural Sciences and Research Council of Canada and National Institutes of Health grant NIBIB T32 EB001628.
