# Supplementary material for: Auditory and reward structures reflect the pleasure of musical expectancies during naturalistic listening
Source: Front Neurosci. 2023 Oct 19;17:1209398. doi: 10.3389/fnins.2023.1209398 (PMC10625409; doi:10.3389/fnins.2023.1209398)
Supplement: Supplementary file 1 [file Data_Sheet_1.DOCX]

Supplementary Material

**Supplementary Table 1.** Stimulus details regarding the title, composer, and year of each excerpted piece; the approximate time window, key, and meter of its excerpt; and the mean duration-weighted information content (mDW-IC) and mean duration-weighted entropy (mDW-Ent) of each stimulus.

| **Piece** | **Composer** | **Year** | **Excerpt Time (approximate)** | **Key** | **Meter** | **mDW-IC** | **mDW-Ent** |
| --- | --- | --- | --- | --- | --- | --- | --- |
| Streams of Kilnaspig | Irish Traditional | Unknown | 0:00 – 0:30 | G Major | Compound Duple | 2.34 | 3.62 |
| Eighteen Studies for the Flute, Op. 41, No. 11 | Joachim Andersen | 1891 | 1:30 – 2:00 | F Major | Simple Duple | 2.99 | 2.23 |
| When This Cruel War is Over | American Traditional | 1863 | 1:00 – 1:30 | Bb Major | Simple Duple | 3.71 | 3.86 |
| Seven Variations on a Theme from Silvana, J. 128, Op. 33, Var. 7 | Carl Maria von Weber | 1854 | 8:00 – 8:30 | Bb Major | Compound Duple | 3.89 | 2.87 |
| 12 Fantasias for Solo Flute, No. 3, Vivace | Georg Philipp Telemann | 1733 | 0:45 – 1:15 | B Minor | Simple Duple | 3.93 | 2.64 |
| Eighteen Studies for the Flute, Op. 41, No. 18 | Joachim Andersen | 1891 | 0:50 – 1:20 | F Minor | Compound Duple | 4.04 | 2.60 |
| 12 Fantasias for Solo Flute, No. 3, Vivace | Georg Philipp Telemann | 1733 | 0:10 – 0:40 | B Minor | Simple Duple | 4.08 | 2.45 |
| Orchestral Suite No. 2 in B minor, BWV 1067 | Johann Sebastian Bach | 1739 | 2:45 – 3:15 | B Minor | Simple Duple | 4.52 | 3.95 |
| Eighteen Studies for the Flute, Op. 41, No. 1 | Joachim Andersen | 1891 | 0:45 – 1:15 | C Major | Simple Duple | 4.97 | 3.60 |
| Five Divertimentos, K. 439b, No. 2, mvmt. 4 | Wolfgang Amadeus Mozart | 1785 | 0:50 – 1:20 | C Major | Simple Triple | 5.00 | 3.12 |
| Gavotte | François-Joseph Gossec | Unknown | 0:00 – 0:30 | C Major | Simple Duple | 5.05 | 2.31 |
| Seven Variations on a Theme from Silvana, J. 128, Op. 33, Theme | Carl Maria von Weber | 1854 | 0:00 – 0:30 | Bb Major | Compound Duple | 5.30 | 3.76 |
| Drei Fantasiestücke, Op. 73, No. 1 | Robert Schumann | 1849 | 0:30 – 1:00 | A Minor | Simple Duple | 5.35 | 4.05 |
| Five Divertimentos, K. 439b, No. 2, mvmt. 4 | Wolfgang Amadeus Mozart | 1785 | 3:50 – 4:20 | G Major | Simple Triple | 5.47 | 3.54 |
| 35 Exercises for Flute, Op. 33, No. 3 | Ernesto Koehler | 1880s | 1:00 – 1:30 | F Major | Simple Triple | 5.54 | 4.01 |
| Eighteen Studies for the Flute, Op. 41, No. 6 | Joachim Andersen | 1891 | 1:00 – 1:30 | B Minor | Simple Triple | 5.57 | 4.09 |
| Carmen Suite No. 1, Aragonaise | Georges Bizet | 1882 | 0:45 – 1:15 | D Minor | Simple Triple | 5.61 | 3.65 |
| Orchestral Suite No. 2 in B minor, BWV 1067 | Johann Sebastian Bach | 1739 | 0:00 – 0:30 | B Minor | Simple Duple | 5.61 | 3.52 |
| 35 Exercises for Flute, Op. 33, No. 15 | Ernesto Koehler | 1880s | 0:00 – 0:30 | E Major | Simple Duple | 5.62 | 3.62 |
| Drei Fantasiestücke, Op. 73, No. 1 | Robert Schumann | 1849 | 1:15 – 1:45 | A Minor | Simple Duple | 5.63 | 3.97 |
| Eighteen Studies for the Flute, Op. 41, No. 10 (practice) | Joachim Andersen | 1891 | 0:00 – 0:30 | C# Minor | Compound Duple | 5.65 | 4.13 |
| 35 Exercises for Flute, Op. 33, No. 10 | Ernesto Koehler | 1880s | 0:00 – 0:30 | D Major | Simple Duple | 5.80 | 4.16 |
| Study No. 1 in C Major, Op. 131 | Giuseppe Gariboldi | 1900 | 0:00 – 0:30 | C Major | Simple Duple | 5.92 | 3.81 |
| Flute Concerto No. 2 in G minor, RV439 “La notte” | Antonio Vivaldi | 1729 | 10:00 – 10:30 | C Minor | Simple Duple | 5.93 | 3.63 |
| Dolly Suite Op. 56, No. 1 | Gabriel Fauré | 1893 | 0:10 – 0:40 | G Major | Simple Duple | 5.97 | 4.19 |
| Flute Concerto No. 2 in G minor, RV439 “La notte” | Antonio Vivaldi | 1729 | 9:15 – 9:45 | G Minor | Simple Duple | 6.05 | 3.83 |
| Solo de Concours | André Messager | 1899 | 4:00 – 4:30 | Bb Major | Simple Duple | 6.08 | 4.22 |
| Eighteen Studies for the Flute, Op. 41, No. 6 | Joachim Andersen | 1891 | 0:00 – 0:30 | B Minor | Simple Triple | 6.09 | 4.07 |
| Student Instrumental Course: Flute Student, Level II book: pg. 12 exercise no. 2 | Douglas Steensland, Fred Weber | 2000 | 0:10 – 0:40 | Ab Major | Simple Duple | 6.09 | 4.11 |
| Fantaisie, Op. 79 | Gabriel Fauré | 1898 | 0:30 – 1:00 | E Minor | Simple Triple | 6.39 | 4.22 |
| 12 Fantasias for Solo Flute, No. 10, Dolce | Georg Philipp Telemann | 1733 | 1:57 – 2:27 | G Minor | Simple Duple | 6.39 | 3.02 |
| 12 Fantasias for Solo Flute, No. 5, Allegro | Georg Philipp Telemann | 1733 | 0:37 – 1:17 | C Major | Simple Triple | 6.49 | 3.69 |
| 35 Exercises for Flute, Op. 33, No. 2 | Ernesto Koehler | 1880s | 0:07 – 0:37 | G Major | Simple Duple | 6.61 | 3.79 |
| 12 Fantasias for Solo Flute, No. 10, Presto | Georg Philipp Telemann | 1733 | 2:45 – 3:15 | F# Minor | Simple Triple | 7.09 | 4.10 |
| Eighteen Studies for the Flute, Op. 41, No. 8 | Joachim Andersen | 1891 | 1:30 – 2:00 | F# Minor | Simple Triple | 7.27 | 4.19 |
| Con Alma | Dizzy Gillespie | 1954 | 1:15 – 1:45 | Ab Major | Simple Duple | 7.64 | 4.03 |
| 35 Exercises for Flute, Op. 33, No. 11 | Ernesto Koehler | 1880s | 1:00 – 1:30 | A Minor | Compound Duple | 7.84 | 4.65 |
| Syrinx | Claude Debussy | 1913 | 2:15 – 2:45 | Bb Minor | Simple Triple | 7.86 | 3.95 |
| Orchestral Suite No. 2 in B minor, BWV 1067 | Johann Sebastian Bach | 1739 | 3:45 – 4:15 | E Minor | Simple Duple | 8.05 | 4.50 |
| Nocturnes, Op. 37, No. 1 | Frédéric Chopin | 1839 | 0:30 – 1:00 | C Minor | Simple Duple | 8.08 | 4.41 |
| Seven Early Songs, Die Nachtigall | Alban Berg | 1907 | 0:30 – 1:00 | A Major | Simple Triple | 8.19 | 3.47 |
| Les Folies d’Espagne, Nos. 7 and 8 | Marin Marais | 1701 | 0:10 – 0:40 | E Minor | Simple Triple | 8.60 | 2.84 |
| Nocturnes, Op. 37, No. 1 | Frédéric Chopin | 1839 | 0:00 – 0:30 | C Minor | Simple Duple | 8.66 | 4.30 |
| Les Folies d’Espagne, No. 5 | Marin Marais | 1701 | 0:00 – 0:30 | E Minor | Simple Triple | 9.47 | 3.50 |
| Le Rossignol en Amour | François Couperin | 1722 | 1:45 – 2:15 | G Major | Simple Triple | 9.56 | 3.84 |
| Citygate/Rumble | Chick Corea | 1986 | 1:00 – 1:30 | Db Major | Simple Duple | 10.74 | 3.78 |
| Alone Together | Arthur Schwartz | 1932 | 0:45 – 1:15 | D Minor | Simple Duple | 10.92 | 3.85 |
| Seven Early Songs, Traumgekrönt | Alban Berg | 1908 | 0:30 – 1:00 | G Minor | Simple Duple | 11.14 | 4.08 |
| Les Folies d’Espagne, No. 1 (practice) | Marin Marais | 1701 | 0:00 – 0:30 | E Minor | Compound Triple | 11.27 | 4.47 |
| First Rhapsody | Claude Debussy | 1910 | 0:30 – 1:00 | F# Minor, E Minor | Simple Duple | 11.31 | 4.51 |
| Syrinx | Claude Debussy | 1913 | 0:00 – 0:30 | Bb Minor | Simple Triple | 14.94 | 3.90 |
| Mei | Kazuo Fukushima | 1962 | 0:37 – 1:07 | Atonal | Simple Duple | 16.51 | 4.61 |

**
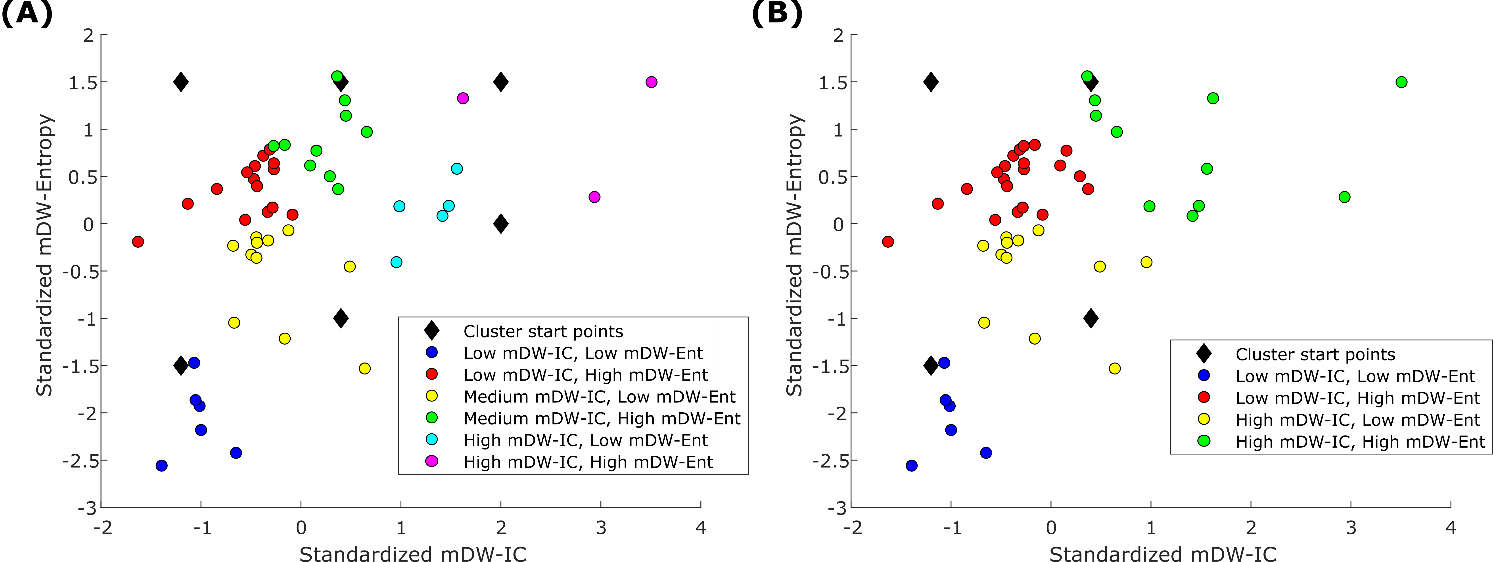
**

**Supplementary Figure 1.** Clustering of stimuli into **(A)** six categories for behavioral analysis and **(B)** four groupings for fMRI analysis. In each case, the black diamonds signify the start points for each cluster, and the colored circles indicate the final k-means assignment of each stimulus. The x and y axes show, respectively, the surprise (i.e., standardized mean duration-weighted information content, or mDW-IC) and uncertainty (i.e., standardized mean duration-weighted entropy, or mDW-Entropy) of each stimulus.

**
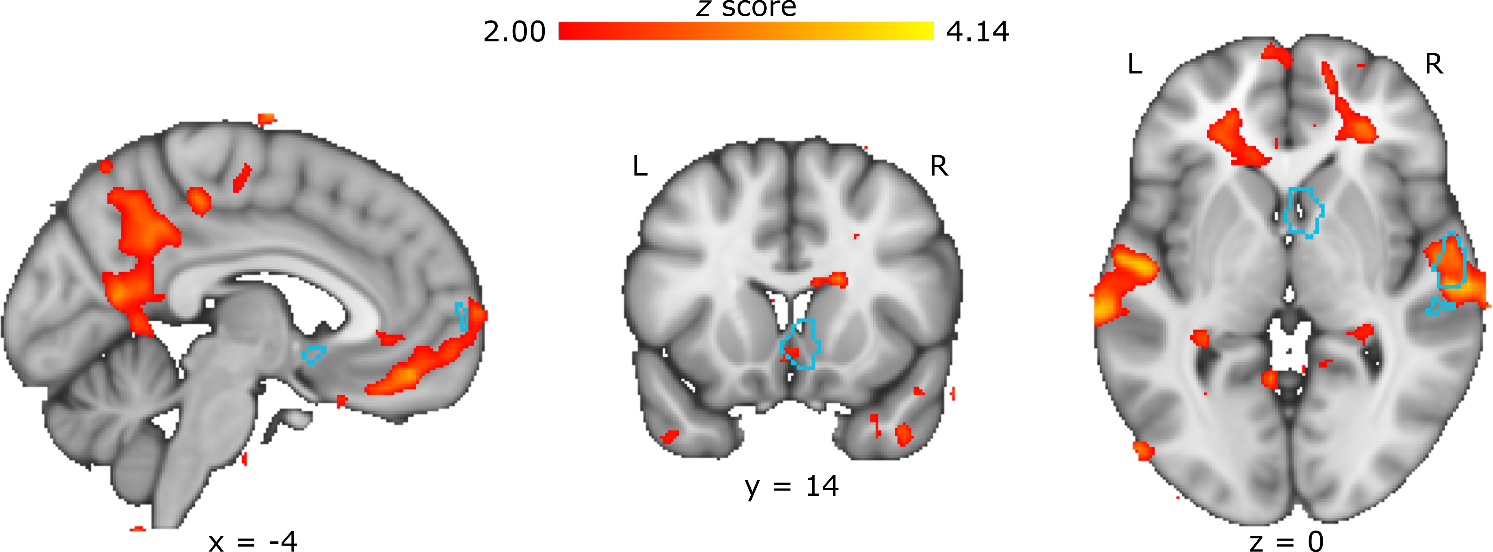
**

**Supplementary Figure 2.** Neural effects of surprise ✕ liking interactions, after accounting for the main effects of surprise and liking. Whole-group interactions between surprise (operationalized as mean duration-weighted information content, or mDW-IC) and liking ratings covaried with BOLD activity in a number of regions, including the R STG and the VS, even when controlling for the main effects of surprise and liking (cf. Figure 3A). Data are visualized in MNI space at an uncorrected threshold of *z* ≥ 2 in a red-to-yellow color scale, while the *a priori* regions of interest (ROIs) are outlined in light blue. L = left. R = right.


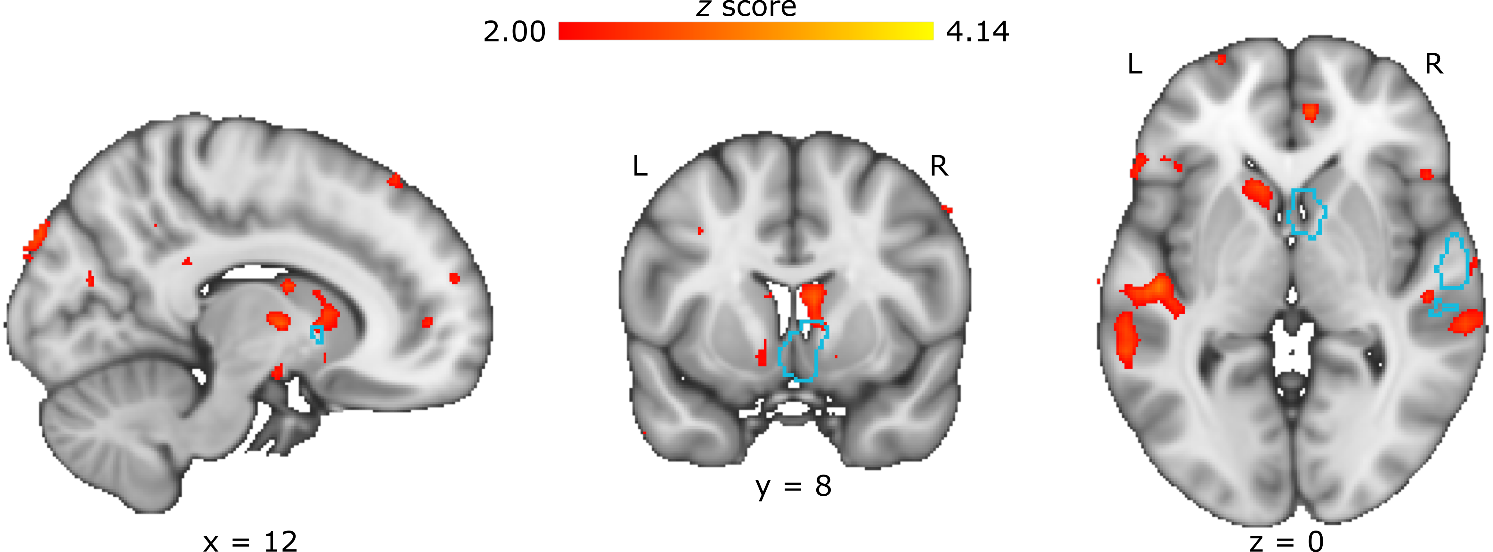


**Supplementary Figure 3.** Neural effects of surprise ✕ uncertainty interactions, after accounting for the main effects of surprise and uncertainty. Whole-group interactions between surprise (operationalized as mean duration-weighted information content, or mDW-IC) and uncertainty (operationalized as mean duration-weighted entropy, or mDW-Entropy) covaried with BOLD activity in a number of regions, including the VS, even when controlling for the main effects of surprise and uncertainty (cf. Figure 4A). Data are visualized in MNI space at an uncorrected threshold of *z* ≥ 2 in a red-to-yellow color scale, while the *a priori* regions of interest (ROIs) are outlined in light blue. L = left. R = right


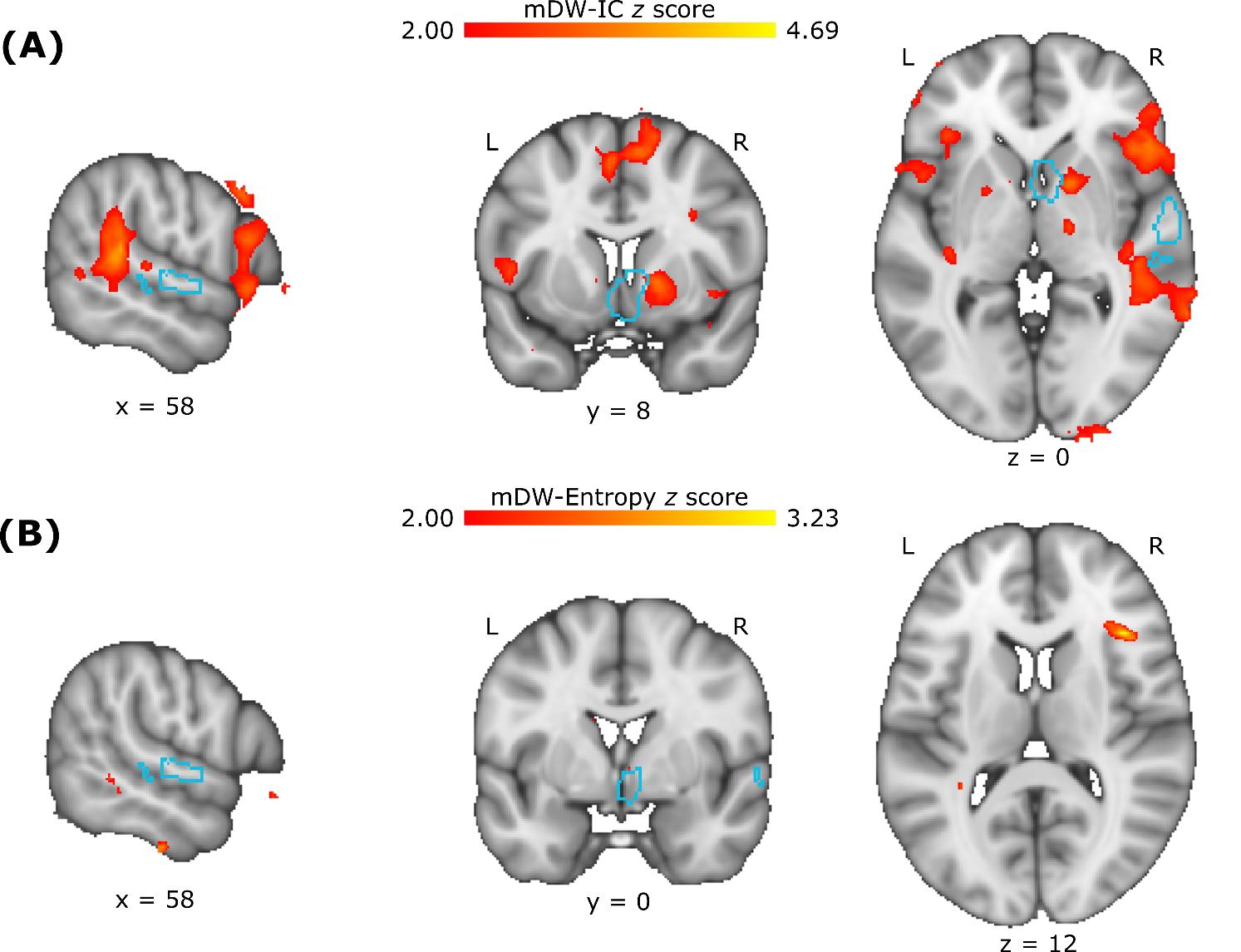


**Supplementary Figure 4.** Main effects of **(A)** surprise (operationalized as mean duration-weighted information content, or mDW-IC) and **(B)** uncertainty (operationalized as mean duration-weighted entropy, or mDW-Entropy). Data are visualized in MNI space at an uncorrected threshold of *z* ≥ 2 in a red-to-yellow color scale, while the *a priori* regions of interest (ROIs) are outlined in light blue. L = left. R = right.
